# Role of Mechanical Cues in Cell Differentiation and Proliferation: A 3D Numerical Model

**DOI:** 10.1371/journal.pone.0124529

**Published:** 2015-05-01

**Authors:** Seyed Jamaleddin Mousavi, Mohamed Hamdy Doweidar

**Affiliations:** 1 Group of Structural Mechanics and Materials Modeling (GEMM), Aragón Institute of Engineering Research (I3A), University of Zaragoza, Zaragoza, Spain; 2 Mechanical Engineering Department, School of Engineering and Architecture (EINA), University of Zaragoza, Zaragoza, Spain; 3 Centro de Investigación Biomédica en Red en Bioingeniería, Biomateriales y Nanomedicina (CIBER-BBN), Zaragoza, Spain; University of Zurich, SWITZERLAND

## Abstract

Cell differentiation, proliferation and migration are essential processes in tissue regeneration. Experimental evidence confirms that cell differentiation or proliferation can be regulated according to the extracellular matrix stiffness. For instance, mesenchymal stem cells (MSCs) can differentiate to neuroblast, chondrocyte or osteoblast within matrices mimicking the stiffness of their native substrate. However, the precise mechanisms by which the substrate stiffness governs cell differentiation or proliferation are not well known. Therefore, a mechano-sensing computational model is here developed to elucidate how substrate stiffness regulates cell differentiation and/or proliferation during cell migration. In agreement with experimental observations, it is assumed that internal deformation of the cell (a mechanical signal) together with the cell maturation state directly coordinates cell differentiation and/or proliferation. Our findings indicate that MSC differentiation to neurogenic, chondrogenic or osteogenic lineage specifications occurs within soft (0.1-1 kPa), intermediate (20-25 kPa) or hard (30-45 kPa) substrates, respectively. These results are consistent with well-known experimental observations. Remarkably, when a MSC differentiate to a compatible phenotype, the average net traction force depends on the substrate stiffness in such a way that it might increase in intermediate and hard substrates but it would reduce in a soft matrix. However, in all cases the average net traction force considerably increases at the instant of cell proliferation because of cell-cell interaction. Moreover cell differentiation and proliferation accelerate with increasing substrate stiffness due to the decrease in the cell maturation time. Thus, the model provides insights to explain the hypothesis that substrate stiffness plays a key role in regulating cell fate during mechanotaxis.

## Introduction

Cell differentiation, proliferation, apoptosis and migration play an important role in the early stages of the tissue regeneration process. The ability of a stem cell to differentiate into different cell types allows it to generate different tissues. For instance, mesenchymal stem cells (MSCs) have the ability to differentiate into fibroblasts, chondrocytes, osteoblasts, neuronal precursors, adipocytes and many others [1–4]. Although, on the one hand, the multi-lineage differentiation potential of stem cells is an advantage, on the other hand, it can be a disaster if they differentiate at the wrong time, at an undesirable place or to an inappropriate cell type. This may lead to a pathophysiologic state or non-functional tissue construction. To overcome such abnormalities, stem cells have been particularized in such a way as to differentiate in response only to appropriate biological cues. Therefore, although cell is able to undergo differentiation, proliferation and/or death due to other signals such as chemotaxis our intention here is to study it from mechanotactic viewpoint.

Cell differentiation and proliferation are governed by a combination of chemical [5] and mechanical [6, 7] cues, although biologists have frequently reported that other cues such as growth factors and cytokines may be involved in the regulation of stem cell differentiation [5, 8]. Recent observations have demonstrated that cell differentiation and proliferation can be significantly influenced by mechanical cues [6, 9]. Experimental studies have shown that mechanical factors, including substrate stiffness, nanotopography of the adhesion surface, mechanical forces, fluid flow and cell colony sizes can direct stem cell fate even in the absence of biochemical factors [3, 4, 7]. Many experimental studies [1, 2, 4, 6, 7, 9–11] have been dedicated to investigating the effect of mechanical cues on cell differentiation and proliferation in tissue regeneration. For instance, Pauwels [11] mentioned that distortional shear stress is a specific stimulus for MSCs to differentiate into fibroblasts for fibrous tissue generation. Hydrostatic compression is a specific stimulus for MSCs to differentiate into chondrocytes in cartilage formation while MSCs differentiate into the osteogenic pathway (ossification) only when the strain felt by the cell is below a defined threshold.

Cells actively sense and react to their micro-environment mechanical conditions (mechano-sensing) through their focal adhesions [4, 6, 7, 9, 12, 13]. For instance, it has been observed that the variation of matrix stiffness from soft to relatively rigid can direct MSC fate [1, 2, 10]. Engler et al. [1] investigated, for the first time, the key role of matrix stiffness on the fate of human MSCs (hMSCs). To study the influence of various matrix stiffnesses on hMSCs, they built artificial matrices ranging from soft to rigid for surface cell attachment. They inferred that matrix stiffness dictates hMSC commitment: cells cultured on soft substrates comparable with brain tissue (a stiffness of 0.1–1 kPa) generated neuronal precursors; matrices with intermediate stiffness resembling the elasticity of muscle tissue (a stiffness of 8–17 kPa) induced myogenic commitment while relatively hard matrices mimicking collagenous bone (a stiffness of 25–40 kPa) committed to an osteogenic lineage specification. The effect of substrate stiffness on mouse MSC lineage specification has also been studied by Huebsch et al. [2] within 3D substrates which are physiologically more a relevant environment as cell substrate. They showed that matrix stiffness plays a significant role in MSC lineage specification where adipogenic commitment was seen in relatively softer micro-environments (a stiffness of 2.5–5.0 kPa) while osteogenic specification predominated in substrates with intermediate elasticity (a stiffness of 11–30 kPa). Their findings indicate that the effect of matrix stiffness on cell phenotype in 3D matrices is generally consistent with 2D experimental observations [1, 10]. Besides, matrix stiffness controls the proliferation of the self-renewal of adult stem cells. For instance, muscle stem cells cultured on intermediate substrates resembling the elasticity of muscle tissue have proliferation potential while they are unable to proliferate on rigid substrates [10].

The signaling mechanisms by which micro-environment stiffness controls cell lineage is still an open question. Many mechano-biological models have been developed to describe cell differentiation during fracture healing [14–21]. For example, Stops et al. [21] simulated cell differentiation and proliferation in a collagen-glycosaminoglycan scaffold subjected to mechanical strain and perfusive fluid flow. They assumed that the responses of the representative cells depend on the level of scaffold strain and the inlet fluid velocity. They demonstrated that according to MSC differentiation patterns, specific combinations of scaffold strains and inlet fluid flows cause phenotype assemblies dominated by single cell types. Besides, Kang et al. [20] developed a model to simulate bone fracture healing corresponding to cell differentiation, proliferation and apoptosis. Their model is formulated based on the cell density of each phenotype assuming that cell differentiation and proliferation can be modulated according to the magnitude and frequency of the mechanical stimuli. According to their numerical results, the bone healing process can be improved when the magnitude and frequency of the mechanical stimuli are employed as controlling factors of cell proliferation.

All of the numerical models mentioned above are able to predict the general patterns of tissue differentiation due to external mechanical stimuli, confirmed by experimental observations of mechano-regulated tissue differentiation. Although defining a general patterns of cell differentiation for the tissue repairing is a challenging issue, as previously discussed, cell differentiation and proliferation can be triggered by mechano-sensing process and cell substrate interaction during cell migration [1, 2, 10]. To the best of our knowledge, there is no numerical model that considers cell differentiation and proliferation based on mechano-sensing process during cell migration. In previous works presented by the same authors [22–24], a numerical model was developed to simulate cell migration in substrates with different effective cues. The main purpose of the present work is to extend the previous model to study the influence of substrate mechanical conditions on cell differentiation, proliferation and apoptosis during migration.

## Model formulation

A discrete finite element approach has been chosen to formulate cell migration, differentiation, proliferation and apoptosis in defined substrates. This approach provides flexibility in the definition of migration direction without the need to remesh the substrate and allows both deterministic and stochastic modelling of cell behaviour [24–26].

### Cell migration

#### Stress transmitted by each individual cell to the ECM

During cell translocation, the actin cytoskeleton (CSK) controls the driving forces at the cell front while the microtubule network regulates the rear retraction of the cell [27–29]. Active stress generated by actin filaments and myosin II, active cellular elements, basically depends on the maximum, *ϵ*_max_, and the minimum, *ϵ*_min_, internal cell strains. Besides, passive stress is related to the microtubules and the cell membrane, passive cellular elements [24, 26]. The cell stress which is transmitted to the ECM can be defined as the sum of the passive and active stresses by [24, 30]

$$\begin{array}{c} \sigma_{\text{cell}}=\{\begin{array}{cc} K_{\text{pas}}\epsilon_{\text{cell}} & \epsilon_{\text{cell}}<\epsilon_{\text{min}}\;\text{or}\;\epsilon_{\text{cell}}>\epsilon_{\text{max}}\\ \frac{K_{\text{act}}\sigma_{\text{max}}\left(\epsilon_{\text{min}}-\epsilon_{\text{cell}}\right)}{K_{\text{act}}\epsilon_{\text{min}}-\sigma_{\text{max}}}+K_{\text{pas}}\epsilon_{\text{cell}} & \epsilon_{\text{min}}\leq \epsilon_{\text{cell}}\leq \tilde{\epsilon }\\ \frac{K_{\text{act}}\sigma_{\text{max}}\left(\epsilon_{\text{max}}-\epsilon_{\text{cell}}\right)}{K_{\text{act}}\epsilon_{\text{max}}-\sigma_{\text{max}}}+K_{\text{pas}}\epsilon_{\text{cell}} & \tilde{\epsilon }\leq \epsilon_{\text{cell}}\leq \epsilon_{\text{max}} \end{array} \end{array}$$ (1)

where *K*_pas_, *K*_act_, *ϵ*_cell_ and *σ*_max_ denote the stiffness of passive and active cellular elements, the internal strain of the cell and the maximum contractile stress exerted by the actin-myosin machinery, respectively, while $\tilde{\epsilon }=\sigma_{\text{max}}/K_{\text{act}}$.

#### Effective mechanical force

**Fig 1:**
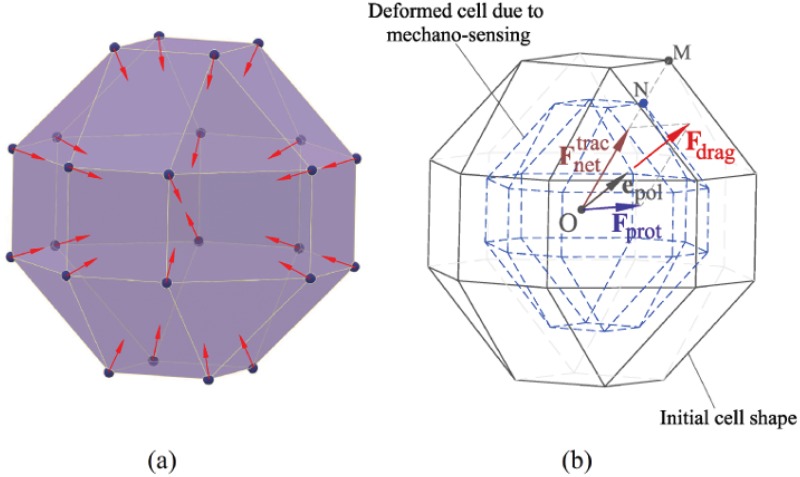
Cell mechanosensing. a- Spherical configuration of the cell in which sensing forces are exerted at each membrane node towards the cell centroid (mechano-sensing process). b- Calculation of the cell internal deformation due to cell mechano-sensing. Deformed cell due to mechano-sensing. **e**_pol_ stands for polarisation direction of the reoriented cell while ${\text{F}}_{\text{net}}^{\text{trac}}$, **F**_prot_ and **F**_drag_ represent the net traction force, protrusion force and drag force, respectively.

During cell migration two main mechanical forces affect the cell body, the traction force and the drag force. The former, which is transmitted to the substrate through integrins, is generated due to the contraction of the actin-myosin apparatus. This force drives the cell body forward and is directly proportional to the cell stress, *σ*_cell_. Representing the cell by a connected group of finite elements, the nodal traction force of the cell can be represented as [24, 25]

$$\begin{array}{c} F_{i}^{\text{trac}}=\sigma_{\text{cell}}S\zeta e_{i} \end{array}$$ (2)

where **e**_*i*_ denotes a unit vector passing through the *i*th node of the cell membrane towards the cell centroid (Fig 1-a). *S* represents the cell membrane area and *ζ* is a dimensionless parameter named “adhesivity” which is directly proportional to the concentration of the ligands at the leading edge of the cell, *ψ*, the total number of available receptors, *n*_*r*_, and the binding constant of the cell integrins, *k*. Therefore, it can be defined as [24, 31]

$$\begin{array}{c} \zeta =kn_{r}\psi \end{array}$$ (3)

Consequently, the net effective traction force on the cell body is calculated as [24]

$$\begin{array}{c} F_{\text{net}}^{\text{trac}}=\sum_{i=1}^{n}F_{i}^{\text{trac}} \end{array}$$ (4)

where *n* is the number of cell membrane nodes.

In contrast, the drag force refers to the force which acts in the opposite direction of the motion of the cell. Our aim here is to specify a velocity-dependent resisting force proportional to the linear viscoelastic character of the substrate. Thus it is assumed that the ECM is a viscoelastic medium [31]. Note that, at microscale, the inertial resistance of the medium can be neglected because it is sufficiently small in comparison with the viscous resistance. Therefore, referring to Stokes’ drag regime, the drag force on a small sphere with radius *r*, moving with velocity *v* within a medium with viscosity *η* can be defined by [24, 31]

$$\begin{array}{c} F_{\text{drag}}=6\;\pi r\eta v \end{array}$$ (5)

#### Protrusion force

To migrate, cells extend local protrusions by exerting a protrusion force to evaluate their surrounding substrate. This refers to the actin polymerization and differs from the cytoskeletal contractile force transmitted to the substrate [22, 25, 31]. It is a random force that causes cells to move along a directed random path towards the effective cue. It is remarkable that the order of the protrusion force magnitude is the same as that of the traction force but with lower amplitude [24, 31–33]. Therefore, it can be described at each time step as

$$\begin{array}{c} F_{\text{prot}}=\kappa F_{\text{net}}^{\text{trac}}e_{\text{rand}} \end{array}$$ (6)

where *κ* is a random number, 0 ≤ *κ* < 1, and $F_{\text{net}}^{\text{trac}}$ denotes the magnitude of the net traction force while **e**_rand_ represents a random unit vector [23, 24]. It is assumed that neither degradation nor remodelling of the ECM occurs during cell migration. As mentioned above, the inertial force is negligible so that the force balance reads

$$\begin{array}{c} F_{\text{net}}^{\text{trac}}+F_{\text{prot}}+F_{\text{drag}}=0 \end{array}$$ (7)

#### Cell deformation and reorientation

For the sake of simplicity a spherical cell shape is considered here (solid line in Fig 1-b). However, any cell shape can be considered using the present model [30]. In the mechano-sensing stage, a cell firstly exerts sensing forces at each finite element node located on the cell membrane towards the cell centroid to probe its surrounding environment. The cell deformation resulting from the mechano-sensing step is shown by dashed lines in Fig 1-b. Therefore, the internal deformation of the cell at each finite element node of the cell membrane can be defined as

$$\begin{array}{c} \epsilon_{\text{cell}}=\frac{\text{MN}}{\text{OM}} \end{array}$$ (8)

Subsequently, referring to Eq 7 the net cell polarisation direction can be calculated by (Fig 1-b)

$$\begin{array}{c} e_{\text{pol}}=-\frac{F_{\text{drag}}}{∥F_{\text{drag}}∥} \end{array}$$ (9)

From Eq 5 and Eq 7 the cell velocity can be defined as

$$\begin{array}{c} v=\frac{∥F_{\text{drag}}∥}{6\;\pi r\eta } \end{array}$$ (10)

Using Eq 10, during time step, *τ*, the translocation vector of the cell through which the cell migrates to locate in a new position can be defined as

$$\begin{array}{c} d=v\tau e_{\text{pol}} \end{array}$$ (11)

### Cell-cell interaction

**Fig 2:**
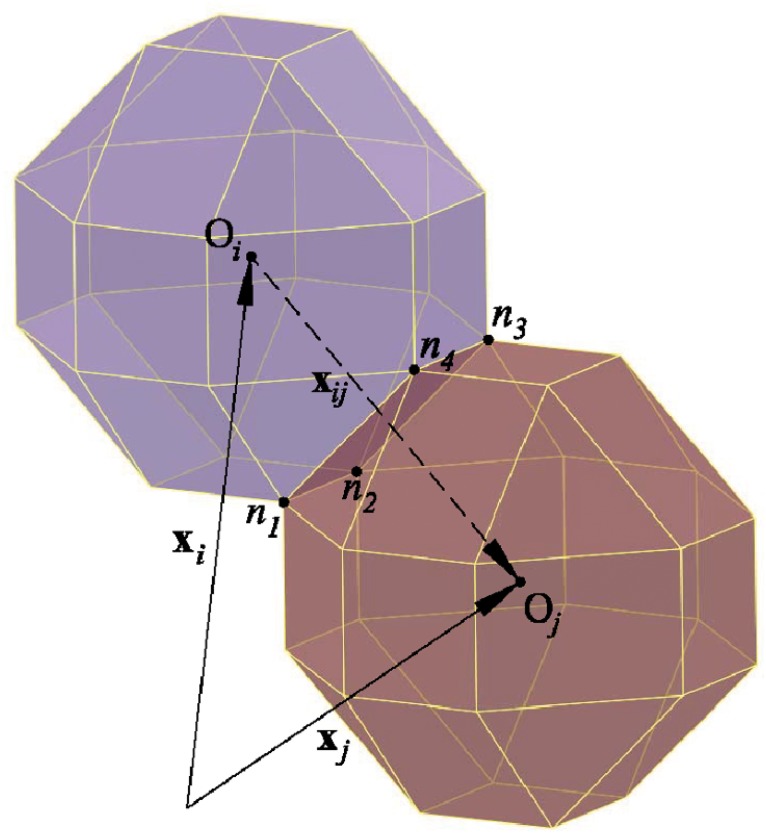
Interaction of two cells in contact. For the assumed cell configuration, two cells can have four common nodes (*n*_1_:*n*_4_). **x**_*i*_ and **x**_*j*_ are position vectors of the *i*th and the *j*th cells, respectively, while **x**_*ij*_ is a vector passing by the centroids of the *i*th and *j*th cells. The distance between their centroids (O_*i*_ and O_*j*_) is equal to or greater than the proposed cell diameter, ‖**x**_*ij*_‖ ≥ 2*r*.

In the presence of two or more cells in a substrate, the traction force, protrusion force, velocity and reorientation of each individual cell can be calculated using the previous formulation. According to Fig 2, a vector passing through the centroid of two cells *i* and *j* can be obtained by

$$\begin{array}{c} x_{ij}=x_{j}-x_{i} \end{array}$$ (12)

where **x**_*i*_ and **x**_*j*_ are position vectors of *i*th and *j*th cells. In reality cells inside a multicellular system do not preserve a spherical shape but deform to be tangent to each other [34]. Here, a useful simplification to avoid interference of two cells is ‖**x**_*ij*_‖ ≥ 2*r*.

For the assumed cell configuration, when two cells come into contact they have a maximum of four common nodes (*n*_1_:*n*_4_ in Fig 2). In vivo, the cell pushes out a pseudopod to get a better sense its environment. Once the cell locates the desired region of the substrate, it pulls itself in the direction of the pseudopod [35]. Therefore, when two or more cells come into contact with each other, the common points of both cells (for instance nodes *n*_1_:*n*_4_ in Fig 2) are not able to send out the pseudopod to the substrate [35, 36]. Therefore, for two or more cells, we assume that cells do not exert any sensing force at their common nodes unless they become separated again due to the random protrusion force. It worth noting that although in such a situation the nodes in contact do not have any role in the mechano-sensing process, the traction forces are not zero in those nodes [22, 24, 26].

### Cell differentiation, proliferation and apoptosis

The importance of sensing the mechanical properties of the ECM has been reported in many experimental studies for different cell types [3, 37, 38]. Cells may respond to the mechanical signals received from their micro-environment by differentiation or apoptosis [39–42]. A specific deformation range experienced by a cell is shown to lead to a specific differentiation [15, 43, 44]. This diversity may arise from differences in their tissue origin, in the magnitude and duration of the mechanical signal sensed by the cell and in the degree of preconditioning. Although the precise effect of mechanical cues on cell apoptosis is still poorly understood, there are experimental works reporting that cell death may occur due to the deformation which a typical cell can bear [39, 41]. Experimental observations of Kearney et al. [41] indicate that tensile strain induced on MSCs mediates cell apoptosis. Cell death depends on the internal stain felt by a cell whereby specific strain ranges in 2D MSC cultures exhibit significant apoptosis while maximal apoptosis occurs in response to 10% tensile cyclic strain when applied continuously. Gladman et al. [39] used an in vitro approach to examine the apoptosis of adult dorsal root ganglion cells. Their finding illustrated that mechanical injury beyond 20% of tensile cyclic strain led to significant neuronal cell death which was also proportional to the duration of the imposed strain.

Let us assume that MSCs are able to differentiate into a certain cell type *i*, where *i* ∈ {*s*, *c*, *l*} represents lineage specifications such as osteoblasts, *s*, chondrocytes, *c*, and neuroblasts, *l*. Mechano-regulation of differentiation is introduced in terms of the mechanical signal (cell internal deformation) in the cell polarisation direction. Deformation of each node located on the cell membrane in the cell polarisation direction can be calculated as

$$\begin{array}{c} \gamma_{i}=e_{\text{pol}}:\epsilon_{i}:e_{\text{pol}}^{\text{T}} \end{array}$$ (13)

where ***ϵ***_*i*_ is the strain tensor of *i*th node located on the cell membrane due to the mechano-sensing process. Therefore, cell internal deformation in the cell polarisation direction can be obtained by

$$\begin{array}{c} \gamma \left(x,t\right)=\sum_{i=1}^{n}\gamma_{i} \end{array}$$ (14)

where *n* is the number of the cell membrane nodes and **x** is the position vector of cell centroid at the time *t*. *γ*_l_ ≤ *γ*(**x**, *t*) ≤ *γ*_u_ varies spatially and temporally during cell migration [15, 20, 45] where *γ*_l_ and *γ*_u_ are lower and upper bounds of cell internal deformations leading to cell differentiation, respectively.

Based on experimental observations [12, 46, 47], not only cell differentiation is mechano-biologically dependent, but it is also time-dependent. For instance, this argument indicates that MSCs [12] and chondrocyte [47] need to be at a certain level of maturity before they undergo differentiation or proliferation. In this context, the cell differentiation depends on a maturation time which is the time that the cell needs to be active in the differentiation stage [20, 46]. The mechanical signals received by a cell can regulate the cell maturation period, being different for each cell type. Although stronger mechanical signals (less internal deformation) can decrease the cell maturation time, after cell culture even within substrates producing the strongest mechanical signals, cells need a minimum time period to start differentiation or proliferation [46]. Therefore, we assume that a strong mechanical signal will increase the differentiation rate while it decreases the cell maturation time linearly as

$$\begin{array}{c} t_{\text{mat}}\left(\gamma ,t\right)=t_{\text{min}}+t_{\text{p}}\gamma \left(x,t\right) \end{array}$$ (15)

where *t*_min_ is the minimum time needed by the cell for differentiation and *t*_p_ is a time proportionality. Therefore, beside lineage specifications *i* ∈ {*s*, *c*, *l*}, each cell is also represented via a maturation index (MI) which can be described as

$$\begin{array}{c} \text{MI}=\{\begin{array}{cc} \frac{t}{t_{\text{mat}}} & t\leq t_{\text{mat}}\\ 1 & t>t_{\text{mat}} \end{array} \end{array}$$ (16)

MI = 1 indicates that a cell (MSCs, osteoblasts, chondrocytes and neuroblasts) is completely mature and is prone for differentiation or proliferation in presence of proper mechanical signal. MI = 0 corresponds to young cell, which means that the cell is not yet able to start the differentiation or proliferation process, even if mechanical stimulus is appropriate. We assume that the evolution of cell MI is an irreversible process. This means that once a specific cell phenotype adheres to the substrate during cell migration, the cell MI cannot be reduced except when the cell phenotype changes due to differentiation. It is of interest to mention that although the cell MI is an irreversible parameter during cell migration, the cell maturation time, *t*_mat_, can decrease or increase depending on the mechanical signal strength received by the cell. Considering these previously mentioned conditions, the process of MSC differentiation and apoptosis related to mechanical signals and maturation can be represented by [20]

$$\begin{array}{c} \text{Cell}\;\text{phenotype}=\{\begin{array}{cc} s & \gamma_{\text{l}}<\gamma \leq \gamma_{s}\;\;\&\;\;\text{MI}=1\\ c & \gamma_{s}<\gamma \leq \gamma_{c}\;\;\&\;\;\text{MI}=1\\ l & \gamma_{c}<\gamma \leq \gamma_{\text{u}}\;\;\&\;\;\text{MI}=1\\ \text{apoptosis} & \gamma_{\text{apop}}<\gamma \\ \text{no}\;\text{diffrentiation} & \text{otherwise} \end{array} \end{array}$$ (17)

It should be noted that small strains exerted cyclically on a typical cell may cause fatigue apoptosis of the cell [41] that we have not considered here.

Cell proliferation is the process of producing two daughter cells from a mother. In normal tissues, this generally refers to cells that replenish the tissue by cell growth followed by cell division. Cell proliferation occurs in defined steps including the first growth phase, the synthesis phase, the second growth phase and the mitosis phase, respectively [48, 49]. During the first growth phase, known as the G1 phase, the cell synthesizes a huge content of biological material. As soon as the G1 phase is completed the cell enters the synthesis phase, the S phase, to replicate its DNA. At the end of the S phase it starts the second growth phase, G2, that finally leads to the mitosis phase, the M phase. Subsequently, reorganization of the cell chromosomes is followed by the cell division so that a mother cell is divided into two daughter cells. This is a critical instant because some cells temporarily stop proliferation by entering into the quiescence state which is called the G0 phase [48, 49].

Our objective here is to model the proliferation process using a biologically appropriate method. It is hypothesized that there are no concerns about shortage of oxygen or nutrients for the cells in culture. Therefore, we intend to model the dominant cell division cycle through two main steps. It is assumed that during the G1, S and G2 phases the cell grows and matures such that when the cell maturation is achieved, depending on the strength of the mechanical signal received by the cell, one mature mother cell may enter into the mitosis phase and divide into two non-mature daughters. Thus, in the present model, the cell is either under maturation or in the proliferation phase. In other words, each cell is in the quiescence phase unless it delivers two daughter cells. Accordingly, cell growth can be considered as

$$\begin{array}{c} \text{Cell}\;\text{growth}\;=\;\{\begin{array}{cc} \text{1}\;\text{mother}\;\text{cell}\;\to \;\text{2}\;\text{daughter}\;\text{cells} & \gamma \leq \gamma_{i}^{\text{prof}}\\ & \&\text{MI}=1\\ \text{no}\;\text{cell}\;\text{division} & \text{otherwise} \end{array} \end{array}$$ (18)

where *i* ∈ {*m*, *s*, *c*, *l*} and $\gamma_{i}^{\text{prof}}<\gamma_{\text{u}}$ is the mechanical signal that defines the proliferation limit of the *i*th cell [20]. Here, *m* represents the MSC phenotype. When a mother cell is divided into two daughter cells, it is assumed that one of the daughter cells is located in the same position as the mother cell, ${\text{x}}_{\text{daut}}^{\left(1\right)}={\text{x}}_{\text{moth}}$, while the other is located in the vicinity of the mother cell as

$$\begin{array}{c} x_{\text{daut}}^{\left(2\right)}=x_{\text{moth}}+2re_{\text{rand}} \end{array}$$ (19)

where “moth” and “daut” subscripts denote mother and daughter cells, respectively while **e**_rand_ represents a random unit vector.

Generally, cell proliferation is not coupled tightly to cell differentiation. Rather, cell differentiation and proliferation have sometimes observed to be concurrent but independent processes [50]. For instance, cell proliferation and differentiation occur simultaneously during embryonic development. Although the molecular mechanisms concurrently regulating these two processes remain largely unknown [51]. Therefore, guiding by experimental observations, in the present model if both conditions of cell differentiation and proliferation are simultaneously satisfied (in the case of MSCs) these processes can congruently occur.

## Finite element implementation

**Fig 3:**
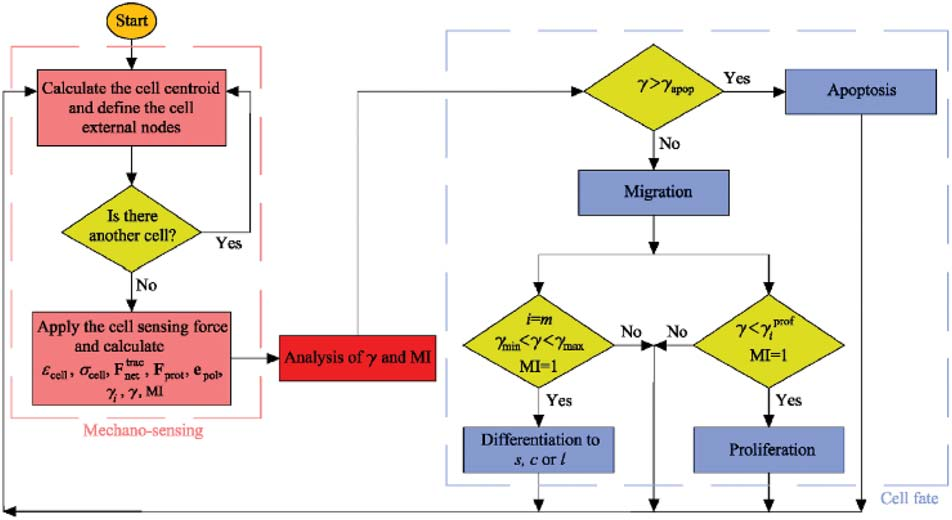
Computational algorithm of cell mechano-sensing and consequent cell fate due to mechanotaxis.

The present model is implemented within the commercial FE software ABAQUS [52] through a coupled user subroutine. The corresponding algorithm is presented in Fig 3.

We have applied the model for several numerical cases where the cell is embedded within a 400×200×200 *μ*m substrate with different ranges of stiffness. It is assumed that there is no external force acting on the substrate and all of the boundary surfaces are considered free. The substrate is meshed by 16,000 regular hexahedral elements and 18,081 nodes while the cell has a constant spherical shape with 24 nodes on its membrane. The calculation time is about one minute for each time step in which each step corresponds to approximately 6 hr of real cell-substrate interaction. The properties of the cells and the substrate are enumerated in Table 1.

**Table 1:** General parameters employed in the model. General parameters employed in the model except where other values are specified.

**Symbol**	**Description**	**Value**	**Ref.**
*ν*	Substrate Poisson ratio	0.3	[42, 53]
*η*	Minimum substrate viscosity	1000 Pa.s	[31, 53]
*K*_pas_	Stiffness of microtubules	2.8 kPa	[54]
*K*_act_	Stiffness of myosin II	2 kPa	[54]
*ϵ*_max_	Maximum strain of the cell	0.9	[24, 33]
*ϵ*_min_	Minimum strain of the cell	-0.9	[24, 33]
*σ*_max_	Maximum contractile stress exerted by actin-myosin machinery	0.1 kPa	[55, 56]
*k*_*f*_ = *k*_*b*_	Binding constant at rear and front of the cell	10^8^ mol^−1^	[31]
*n*_*r*_*f*__	Number of available receptors at the front of the cell	1.5×10^5^	[31]
*n*_*r*_*b*__	Number of available receptors at the back of the cell	10^5^	[31]
*ψ*	Concentration of the ligands at rear and front of the cell	10^−5^ mol	[31]
*t*_min_	Minimum time needed for cell proliferation	4 days	[20, 46]
*t*_p_	Time proportionality	200 days	[20, 46]
*γ*_l_	Lower bound of cell internal deformation leading to osteoblast differentiation	0.005	[20, 57]
*γ*_s_	Upper bound of cell internal deformation leading to osteoblast differentiation	0.04	[20, 57]
*γ*_c_	Upper bound of cell internal deformation leading to chondrocyte differentiation	0.1	[20]
*γ*_u_	Upper bound of cell internal deformation leading to neuroblast differentiation	0.5	
*γ*_apop_	Cell internal deformation leading to cell apoptosis	1	[20]
$\gamma_{m}^{\text{prof}}$	Limit of MSC proliferation	0.2	[20]

## Numerical examples

As discussed before, MSCs can be differentiated into neurogenic, chondrogenic or osteogenic cell types by varying the magnitude of the matrix stiffness to mimic that of the native tissue [1, 2, 10]. However, not all cell types are sensitive to substrate stiffness or have a similar mechano-sensitive response to changes in substrate stiffness [58]. Our objective here is to study the dependency of cell fate on substrate stiffness according to available experimental observations. The model presented here will be used to predict cell fate within soft (0.1–1 kPa), intermediate (20–25 kPa) and hard (30–45 kPa) substrates that are comparable with neurogenic, chondrogenic and osteogenic tissue, respectively. This section provides an insight into the role of substrate stiffness in MSC proliferation in order to examine how cells are able to detect and respond to alterations in the stiffness of their surrounding micro-environment via induction of lineage-specific differentiation. To obtain reliable and consistent results all the numerical cases have been repeated at least 20 times.

### MSC proliferation and differentiation

**Fig 4:**
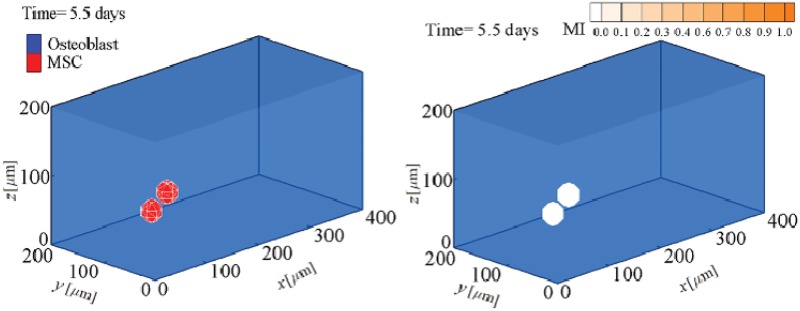
MSC proliferation and differentiation within a substrate of 45 kPa stiffness after 5.5 days. MSC proliferation (see also S1 Video).

**Fig 5:**
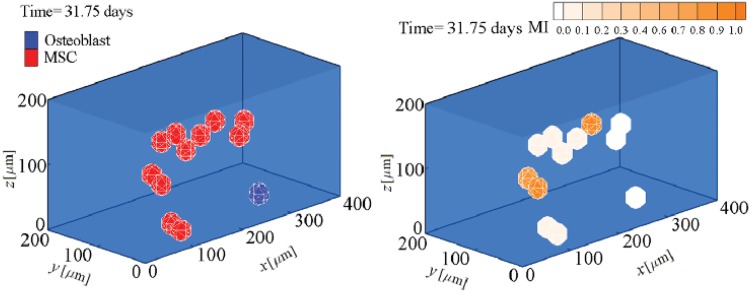
MSC proliferation and differentiation within a substrate of 45 kPa stiffness after 32 days. The first commitment of a mature mother MSC to osteogenic lineage specification (see also S1 Video)

**Fig 6:**
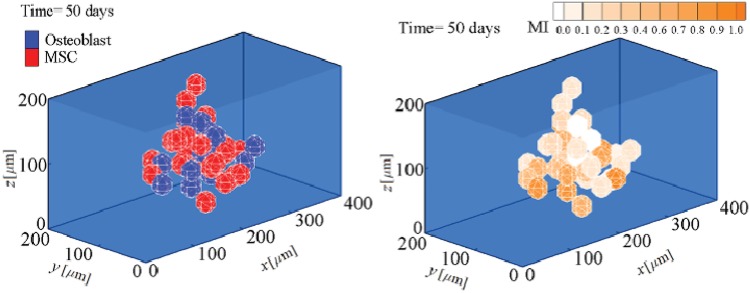
MSC proliferation and differentiation within a substrate of 45 kPa stiffness after 50 days. Continuing differentiation and proliferation of MSCs and osteoblasts (see also S1 Video).

**Fig 7:**
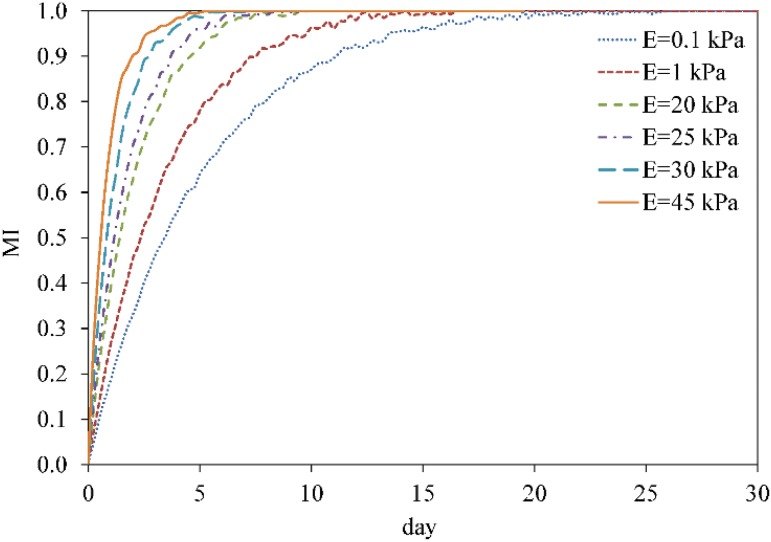
MI of MSCs within substrates of different uniform stiffnesses. *E* represents substrate elasticity modulus.

**Fig 8:**
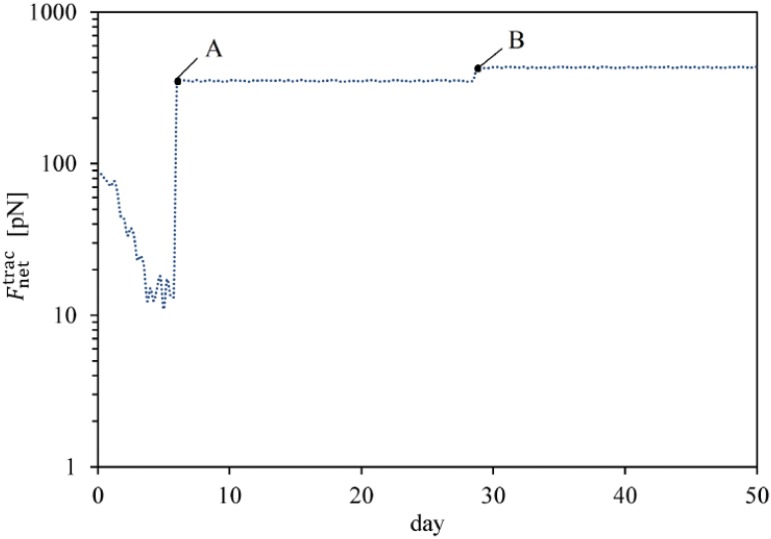
Average cell traction force within a hard substrate of 45 kPa stiffness. Average cell traction force, $F_{\text{net}}^{\text{trac}}$, versus time within a hard substrate of 45 kPa stiffness during MSC proliferation and differentiation. Point A represents the instant of MSC proliferation which causes a considerable jump in the average net traction force while point B is the initial instant of MSC differentiation to osteoblast leading to an enhancement of the average net traction force.

The fate decision of MSCs can be influenced by the microenvironment in which they reside. Their coordinated interactions with the ECM and neighbour cells provide biomechanical signals that direct them to survive, migrate, proliferate or differentiate [3]. Here, using the presented model, we investigate MSC proliferation and differentiation in substrates with different uniform stiffnesses. MSC behavior within a hard substrate of 45 kPa stiffness is represented in Figs 4–6 (results for soft and intermediate substrates are not shown here). Initially, it is assumed that a MSC is located in one of the corners of a hard substrate. Regardless the substrate stiffness, the initial cell tendency is to migrate towards the middle of the substrate where it feels less internal deformation and more stability (discussed previously in [24]). During cell migration over time, the cell becomes mature, however the cell maturation rate depends on the substrate stiffness. After cell maturation (Fig 5), depending on the mechanical signal received by MSC, *γ*, one mature mother MSC may proliferate and deliver two daughter cells in a non-mature state (Fig 6). It is worth noting that an increase in the substrate stiffness decreases the time needed for cell maturation (see Fig 7). This occurs because the increase in the substrate stiffness decreases the internal deformation of the cell which in turn decreases the MSC maturation time (see Eq 15). Accordingly, the shorter maturation times of MSCs, the higher their proliferation rate. This has been suggested by the experimental findings of Evans et al. [59] which revealed that cell growth is increased as a function of substrate stiffness. Besides, during cell migration towards the middle of the substrate, the net traction force generated by the cell decreases which means that the cell can be adhered to the substrate consuming less energy (discussed previously in [23, 24, 26]). Fig 8 demonstrates the average cell traction force versus time within a hard substrate of 45 kPa stiffness during MSC proliferation and differentiation. Point A represents the instant of MSC proliferation (coincident with Fig 4) which causes a considerable jump in the average net traction force. This occurs because upon MSC proliferation, cell-cell interaction causes an asymmetric distribution of the internal cell deformation and subsequently the nodal traction force in the membrane nodes (see [24, 26] for more details about the effect of cell-cell interaction on average traction force). Point B is the instant of MSC differentiation to osteoblast (coincident with Fig 5) leading to an enhancement of the average net traction force which is qualitatively consistent with the observations of Fu et al. [60].

### Fate decision of MSCs in hard substrates (osteogenic tissue)

**Fig 9:**
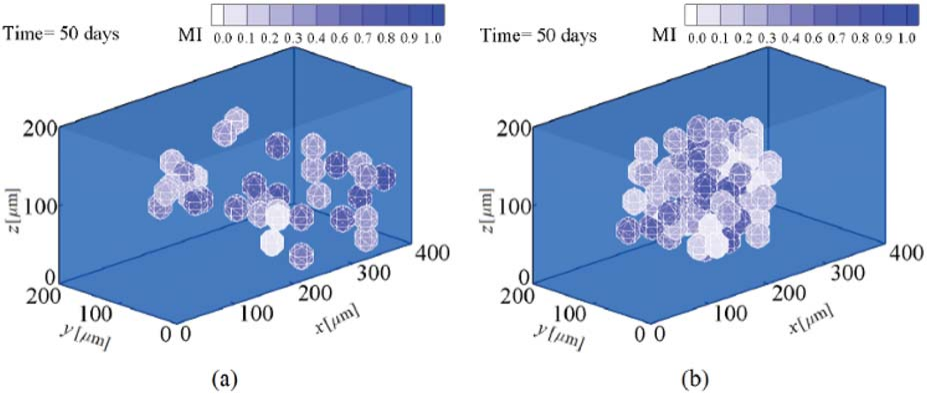
Osteoblast proliferation in hard substrates. a- 30 kPa and b- 45 kPa stiffness (see also S2 Video).

**Fig 10:**
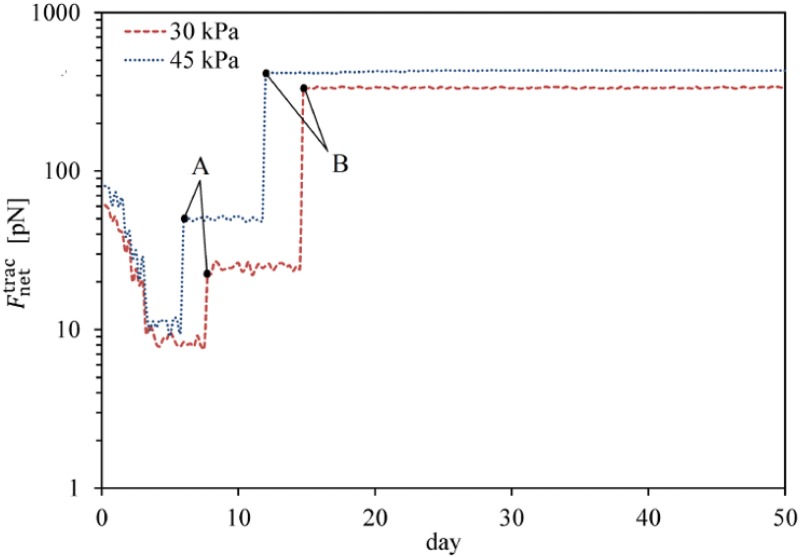
Average cell traction force within a hard substrate of 30 kPa and 45 kPa stiffness. Average cell traction force, $F_{\text{net}}^{\text{trac}}$, versus time within hard substrates during MSC differentiation and osteoblast proliferation. Points A represent the instant of MSC differentiation to osteoblast which instantly causes a traction force increase while points B are the initial instant of osteoblast proliferation causing a jump in the average net traction force.

**Fig 11:**
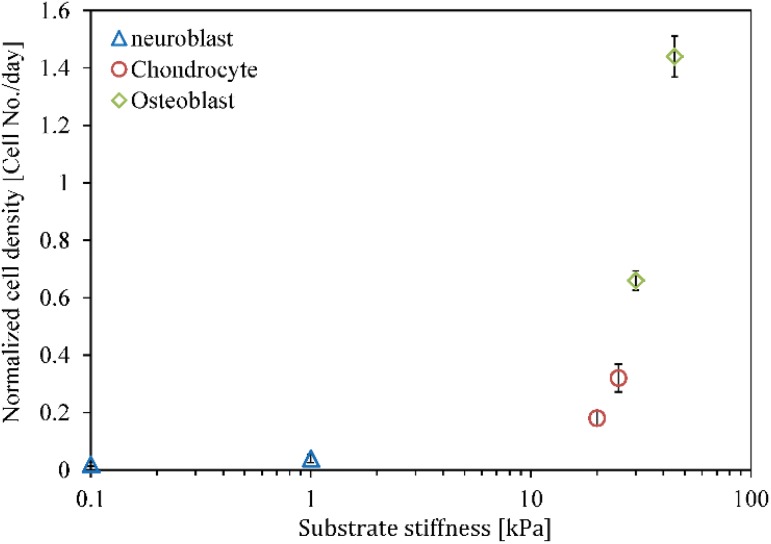
Normalized density of each cell phenotype. Normalized density of each cell phenotype versus substrate stiffness during identical times as a consequence of MSC differentiation and proliferation of each cell phenotype. The error bars represent mean standard deviation of different runs.

This numerical example is designed to study the lineage specification of MSCs in hard substrates. Here, to avoid repetition of the proliferation process of MSCs discussed above, we will present the results starting from the instant of cell differentiation. It is assumed that the stiffness of osteogenic tissue is in the range of 30–45 kPa [61]. In order to investigate the influence of matrix stiffness on osteogenic lineage specification, simulations were repeated for lower (30 kPa) and upper (45 kPa) bounds of the hard substrates. The commitment of MSCs to osteogenic lineage specification in substrates of 30 kPa and 45 kPa stiffnesses is presented in Fig 9-a and 9-b, respectively. During migration, the cell gradually matures and once the MSC is completely mature (MI = 1) it differentiates into osteoblast within both hard substrates. Osteoblast lineage specification of MSCs within a substrate with a stiffness equivalent to that of osteogenic tissue is supported by the experimental observations of Engler [1] and Huebsch [2]. The MSC is mature and differentiates into osteoblast within a substrate of 30 kPa stiffness after ∼7 days while it becomes mature and differentiates within a substrate of 45 kPa stiffness after ∼5.5 days. Therefore, consistent with the findings of Fu et al. [60], an increase in the substrate stiffness expedites MSC differentiation to osteoblast. In addition, this is in agreement with the observations of Evans et al. [59] who show that terminal osteogenic differentiation of Embryonic SCs (ESCs) is enhanced on stiff substrates compared with soft substrates. Although cell migration towards the middle of the substrate causes the net traction force to decrease [24, 26], MSC differentiation to osteoblast instantly leads to a greater magnitude of the net traction force (points A in Fig 10). A strong correlation between the traction force and the ultimate lineage specification of MSCs has been observed by Fu et al. [60], while their observations indicate that the osteogenic lineage specification of MSCs demonstrates higher traction force than that of the progenitor MSCs. This can be attributed to mechanical coupling between the extracellular matrix (ECM) and internal CSK organization, according to the suggestion of Zemel et al. [62] which indicates that a perfect alignment of stress fibers in the direction of the cell polarisation occurs when the cell and matrix stiffness are similar due to the differentiation of MSCs into osteoblasts. After MSC differentiation to osteoblast within hard substrates, new cell phenotypes can be proliferated depending on the strength of the mechanical signal received by the cell and its maturation state. After MSC differentiation, osteoblast becomes fully mature and starts to proliferate within substrates of 30 kPa and 45 kPa stiffnesses after ∼ 14.5 and ∼ 12 days, respectively. So each new mature osteoblast within substrates of 30 kPa and 45 kPa stiffnesses can be proliferated to many osteoblasts, as shown in Fig 9-a and 9-b, respectively. The normalized density of each cell phenotype versus substrate stiffness is shown in Fig 11 for identical times. Comparing the corresponding Fig 9-a and 9-b and taking into account the results in Fig 11, it can be concluded that, similar to MSC differentiation to osteoblast, the proliferation of osteoblasts is accelerated by an increase in matrix stiffness, due to the decrease in the maturation time. This is in agreement with the findings of Fu et al. [60]. Moreover, during osteoblast proliferation the average magnitude of the net traction force considerably increases (points B in Fig 10), due to cell-cell interaction which causes an asymmetric nodal traction force distribution [24, 26].

### Fate decision of MSCs in intermediate substrates (chondrogenic tissue)

**Fig 12:**
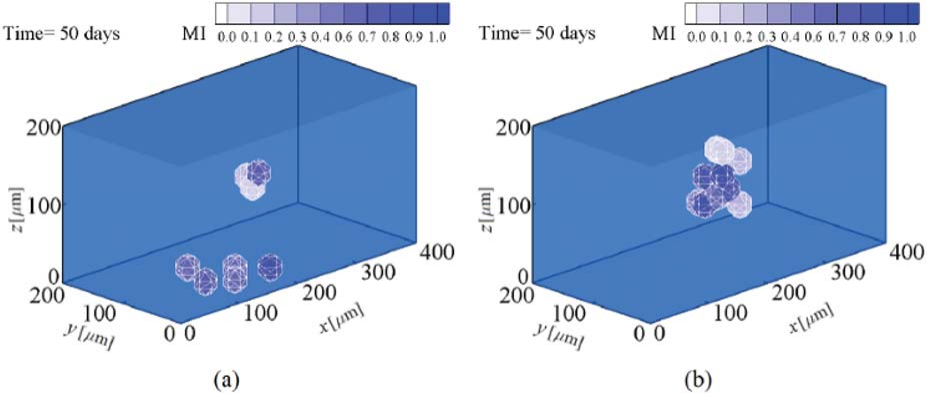
Chondrocyte proliferation in intermediate substrates. a- 20 kPa and b- 25 kPa stiffness (see also S3 Video).

**Fig 13:**
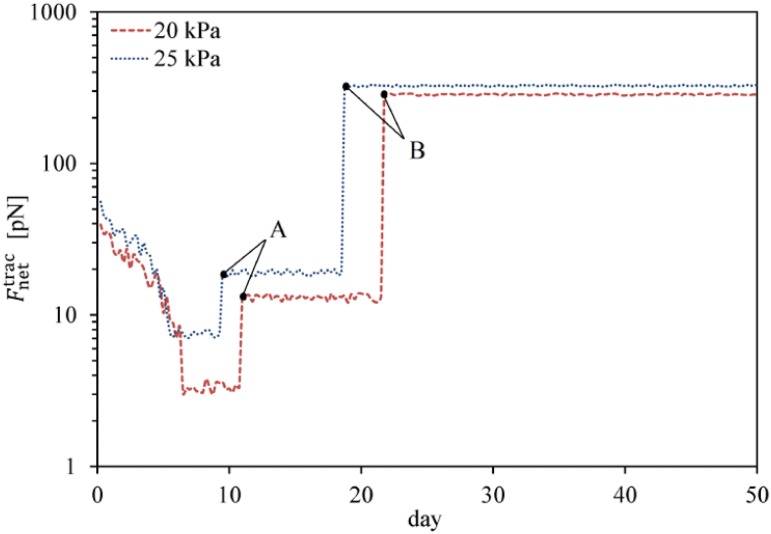
Average cell traction force within intermediate substrates of 20 kPa and 25 kPa stiffness. Average cell traction force, $F_{\text{net}}^{\text{trac}}$, versus time within intermediate substrates during MSC differentiation and chondrocyte proliferation. Point A represents the moment of MSC differentiation to chondrocyte which instantly causes the traction force to increase while point B is the initial moment of chondrocyte proliferation causing a jump in the average net traction force.

To study the lineage specification of MSCs in substrates with intermediate stiffness, simulations are repeated for substrates mimicking the elasticity of chondrogenic tissue. Again, to avoid repetition of the proliferation process of MSCs, the results will be represented starting from the instant of cell differentiation. The stiffness of chondrogenic tissue is assumed to be in the range of 20–25 kPa [61]. MSCs located within substrates of 20 kPa and 25 kPa stiffnesses are completely mature and start to differentiate into chondrocyte after ∼ 10.5 and ∼ 8 days, respectively. So, similar to the previous numerical case, an increase in substrate stiffness in the range of intermediate tissue assists MSC differentiation to chondrocyte. MSC differentiation to chondrocyte in substrates of 20 kPa and 25 kPa stiffnesses is presented in Fig 12-a and 12-b, respectively. This is consistent with the findings of Burke and Kelly [63] indicating that MSC differentiation along either a chondrogenic, osteogenic or adipogenic lineage specification is regulated by the stiffness of the local substrate and the local oxygen tension. According to their results, chondrogenesis of MSCs occurs within matrices that mimic the micro-environmental elasticity of chondrogenic tissue. Furthermore, MSC differentiation to chondrocyte causes the net traction force exerted by the chondrocyte to increase (points A in Fig 13). However its amplification is less than that of the osteoblast traction force within hard substrates. Depending on the maturation state and mechanical signal received by the new cell phenotype, it can be proliferated to many similar cell types. After ∼ 21 and ∼ 18 days, chondrocytes differentiated from MSCs located within substrates of 20 kPa and 25 kPa stiffnesses, respectively, are sufficiently mature to proliferate. As seen in Fig 12-a and 12-b, maturated chondrocytes can be proliferated to many chondrocytes within substrates of 20 kPa and 25 kPa stiffnesses, respectively. Our findings indicate that like the differentiation process, the proliferation of chondrocytes is advanced by an increase in the matrix stiffness in the range of chondrogenic tissue. The chondrocyte density over an identical time period is higher for a substrate with greater stiffness according to the results shown in Figs 11, 12-a and 12-b. In addition, chondrocyte proliferation causes the average magnitude of the net traction force to increase as a consequence of cell-cell interaction (points B in Fig 13).

### Fate decision of MSCs in soft substrates (neurogenic tissue)

**Fig 14:**
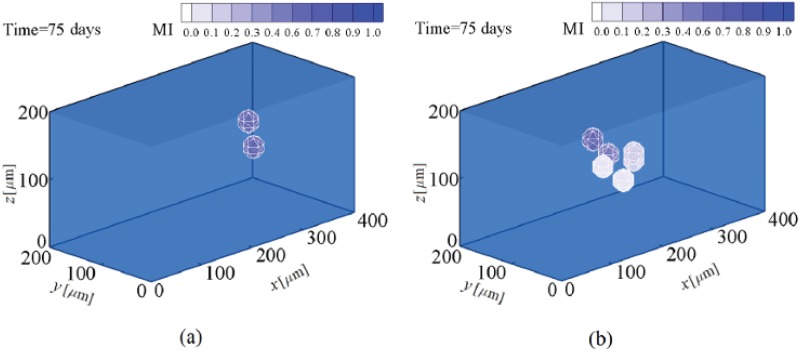
Neuroblast proliferation in soft substrates. a- 0.1 kPa and b- 1 kPa stiffness (see also S4 Video).

**Fig 15:**
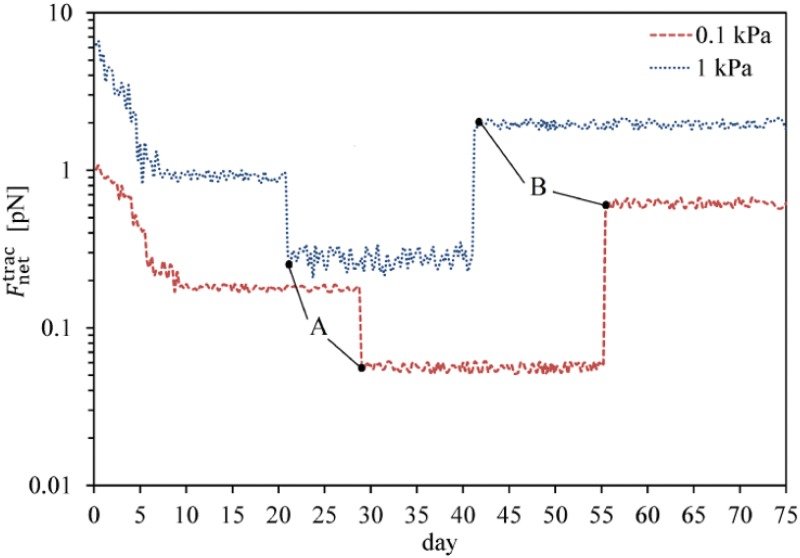
Average cell traction force within soft substrates of 0.1 kPa and 1 kPa stiffness. Average cell traction force, $F_{\text{net}}^{\text{trac}}$, versus time within soft substrates during MSC differentiation and neuroblast proliferation. Points A represent the instant of MSC differentiation to neuroblast which instantly causes the traction force to decrease while points B are the initial instant of neuroblast proliferation causing a jump in the average net traction force.

Experimental observations confirm that neural precursor cells can be obtained from MSCs by culturing them in substrates having a very low stiffness (0.1–1 kPa), comparable to that of the neurogenic tissue [1, 61, 64]. As with the earlier examples, we will represent the results starting from the instant of cell differentiation to avoid repetition of the MSC proliferation process. MSCs located within substrates of 0.1 kPa and 1 kPa stiffnesses are mature and initiate the differentiation process after ∼ 28.5 and ∼ 20.5 days, respectively. Fig 14-a and 14-b represent the cell response to matrix stiffness in substrates of 0.1 kPa and 1 kPa stiffnesses, respectively. The acceleration of MSC differentiation by an increase in the substrate stiffness within relatively soft substrates is supported by the experimental observations of Fu et al. [60] for adipoblasts, differentiating in substrates of 2.5–5 kPa stiffness. Within a neurogenic medium, MSC is more contractile than neuroblast which causes a sudden decrease in the traction force at the instant of MSC differentiation to neuroblast (points A in Fig 15). This is consistent with the findings of Fu et al. [60] for MSC differentiation in soft substrates. After MSC differentiation to neuroblast, a new cell phenotype proliferates depending on its maturation state and the mechanical signal received by the neuroblast. Therefore, MSC differentiation is followed by full maturation of neuroblasts within substrates of 0.1 kPa and 1 kPa stiffnesses after ∼ 57 and ∼ 41 days, respectively. Subsequently, the neuroblast may proliferate to several cells as seen in Fig 14-a and 14-b which indicates that neuroblast proliferation within a substrate of 1 kPa stiffness is quicker than that within a substrate of 0.1 kPa stiffness. The same conclusion can be drawn from the normalized cell density over an identical time period, as shown in Fig 11. This occurs because a higher substrate stiffness advances the instant of cell maturation. In this case, as in the previous ones the cell-cell interaction increases the average magnitude of the net traction force due to neuroblast proliferation (points B in Fig 15).

## Conclusions

The comprehensive signaling mechanisms by which micro-environmental stiffness controls the lineage specification of MSCs is still unknown [3]. Recently, a number of different hypotheses have been proposed about how mechanical signals govern cell fate [3, 15]. To acquire accurate control over cell differentiation and proliferation, it is essential to elucidate and quantify the contribution of mechanical factors to cell response. In this paper, we study the influence of substrate stiffness on cell fate through a new 3D numerical model. This model can be considered as a first step towards the interpretation of existing knowledge about the effect of cellular micro-environment on cell fate. However, we believe that further research on mechanical and physical factor, such as cell shape, topographic changes, external mechanical forces and colony size, can provide a broader understanding of the determination of the cell fate. In this study, we have investigated how a 3D substrate with soft, intermediate and hard stiffnesses influences the differentiation of MSCs towards neurogenic, chondrogenic or osteogenic lineage, respectively. Our findings qualitatively correlated with experimental observations [1, 2, 59, 60, 62] indicate that matrix stiffness can govern the lineage type of MSCs. The traction force generated by a specific cell phenotype can increase (osteoblasts and chondrocytes) or decrease (neuroblast) during differentiation [60]. In contrast, in all cases the proliferation of a typical cell considerably increases the average cell traction force due to the cell-cell interaction that causes an asymmetric distribution of the nodal traction force [22]. In addition, an increase in the matrix stiffness accelerates cell proliferation and differentiation [59]. For a typical cell, internal deformation and focal adhesions developed through integrins are key molecular mechanisms in the mechano-sensing process. For instance, Friedland et al. [65] reported that *α*5*β*1-integrin could switch between relaxed and tensioned states in response to traction forces generated by a cell. Therefore, any changes in the matrix stiffness causes alteration in the cell internal deformation which accordingly regulates the cell differentiation or proliferation. On the other hand, the cell behavior in term of cell differentiation or proliferation depends on the cell maturation. Interestingly, according to Fig 7, cells might require a longer time to become fully mature within soft substrates compared to hard and intermediate substrates. This is consistent with the observations of Hera et al. [64]. Taken together, the results of the model presented here and the earlier experimental observations [1, 2, 59, 60, 62] show that matrix stiffness plays a significant role in controlling the fate decision of MSCs. Accordingly, the present 3D numerical model can successfully predict essential aspects of cell maturation, differentiation, proliferation and apoptosis during regenerative events.

## Supporting Information

S1 Video MSC proliferation, differentiation (LHS) and maturation (RHS) within a substrate of 45 kPa stiffness. MSC proliferates after almost 5.5 days and a mature mother MSC firstly differentiates to osteoblast after approximately 32 days. Afterwards, both MSC and osteoblast continue differentiation and proliferation.
(AVI)

S2 Video Osteoblast proliferates in hard substrates of 30 kPa (LHS) and 45 kPa (RHS) stiffness. (AVI)

S3 Video Chondrocyte proliferates in intermediate substrates of 20 kPa (LHS) and 25 kPa (RHS) stiffness. (AVI)

S4 Video Neuroblast proliferates in soft substrates of 0.1 kPa (LHS) and 1 kPa (RHS) stiffness. (AVI)
